# Targeted Virome Sequencing Enhances Unbiased Detection and Genome Assembly of Known and Emerging Viruses—The Example of SARS-CoV-2

**DOI:** 10.3390/v14061272

**Published:** 2022-06-11

**Authors:** Vasiliki Pogka, Gethsimani Papadopoulou, Vaia Valiakou, Dionyssios N. Sgouras, Andreas F. Mentis, Timokratis Karamitros

**Affiliations:** 1 Laboratory of Medical Microbiology, Department of Microbiology, Hellenic Pasteur Institute, 11521 Athens, Greece; vpoga@pasteur.gr (V.P.); sgouras@pasteur.gr (D.N.S.); mentis@pasteur.gr (A.F.M.); 2 Bioinformatics and Applied Genomics Unit, Department of Microbiology, Hellenic Pasteur Institute, 11521 Athens, Greece; gesthpap@pasteur.gr (G.P.); vanessa.valiakou@gmail.com (V.V.)

**Keywords:** target enrichment, virome sequencing, SARS-CoV-2, COVID-19, NGS diagnostics, emerging viruses, nanopore sequencing

## Abstract

Targeted virome enrichment and sequencing (VirCapSeq-VERT) utilizes a pool of oligos (baits) to enrich all known—up to 2015—vertebrate-infecting viruses, increasing their detection sensitivity. The hybridisation of the baits to the target sequences can be partial, thus enabling the detection and genomic reconstruction of novel pathogens with <40% genetic diversity compared to the strains used for the baits’ design. In this study, we deploy this method in multiplexed mixes of viral extracts, and we assess its performance in the unbiased detection of DNA and RNA viruses after cDNA synthesis. We further assess its efficiency in depleting various background genomic material. Finally, as a proof-of-concept, we explore the potential usage of the method for the characterization of unknown, emerging human viruses, such as SARS-CoV-2, which may not be included in the baits’ panel. We mixed positive samples of equimolar DNA/RNA viral extracts from SARS-CoV-2, coronavirus OC43, cytomegalovirus, influenza A virus H3N2, parvovirus B19, respiratory syncytial virus, adenovirus C and coxsackievirus A16. Targeted virome enrichment was performed on a dsDNA mix, followed by sequencing on the NextSeq500 (Illumina) and the portable MinION sequencer, to evaluate its usability as a point-of-care (PoC) application. Genome mapping assembly was performed using viral reference sequences. The untargeted libraries contained less than 1% of total reads mapped on most viral genomes, while RNA viruses remained undetected. In the targeted libraries, the percentage of viral-mapped reads were substantially increased, allowing full genome assembly in most cases. Targeted virome sequencing can enrich a broad range of viruses, potentially enabling the discovery of emerging viruses.

## 1. Introduction

Emerging and re-emerging viruses appear in a population for the first time or might have existed previously. They are characterized by rapid increments in incidence in geographical regions [1]. Emerging viruses adopt different strategies in order to evade or escape host immune defences [2]. They also exhibit high flexibility in adapting to their current and new hosts, frequently switching between animals and humans [3,4,5,6]. Virtually, all emerging diseases originate from animal populations, while during their adaptation to a new host, they might colonize without causing disease while being able to re-infect another host. During this incubation period, and soon after the emergence of an unknown disease, the accurate and prompt diagnosis of the infectious agent and the development of containment measures, although challenging, is of paramount importance since any delay may result in its full emergence. The new coronavirus SARS-CoV-2, has spread around the world, causing the unprecedented pandemic of COVID-19, although its genome was accurately characterized soon after its emergence in January 2020, in Wuhan province of China. Point-of-care (PoC) testing, the isolation of infected individuals, contact tracing and lockdowns are often the first line of defence in such cases [7,8]. Although the efficacy of containment measures may differ among population groups [9], the groups for which the measures are more effective may vary across populations [10,11].

**Figure 1:**
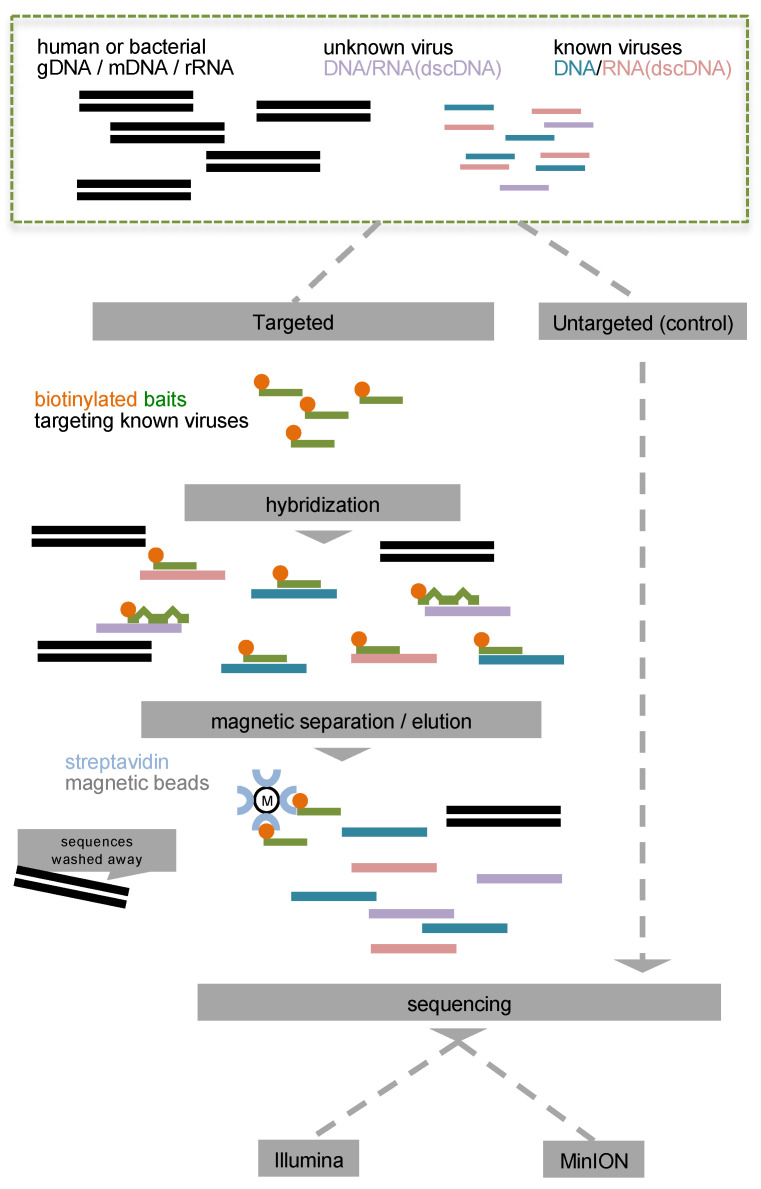
Schematic representation of the study design. Targeted virome library preparation enriches both DNA and RNA viruses as well as unknown viruses (in pink) due to the partial hybridization of the molecular baits (green—orange) to the viral genomes. Targeted and untargeted (control) libraries are sequenced in parallel.

Traditionally, the direct detection of pathogens using molecular diagnostics, such as real-time PCR, microarrays and standard serological assays is considered the “gold standard” for the characterization of an infectious agent. However, these applications may suffer from either discrete or narrow detection ranges—which result in occasionally missing co-infections as only one or a few pathogens are targeted each time—or from low sensitivity and an extended turnaround time (TAT), which is clinically unacceptable. The latter is also the main drawback for the most recently developed metagenomic applications. Making use of the revolutionary technology of next generation sequencing (NGS), metagenomic applications can successfully sequence virtually all genomes present in a clinical sample, define antimicrobial susceptibility or the severity of pathogenesis [12] and have the ability to identify new pathogens. With the exception of robust solutions using the well-standardized sequencing of the 16S gene for bacteria characterization, viral metagenomics, in the setting of a diagnostic laboratory, suffer from technical issues such as the presence of host genomic material, which affects their sensitivity. NGS libraries can be enriched for particular DNA sequences that correspond to genomic targets of interest, depleting irrelevant regions [13]. Target enrichment by hybridization is the method of choice in such cases, as it relies on single stranded oligos, conjugated with biotin, also called molecular “baits” [14]. Following hybridization with the genomic target of interest, streptavidin-coated magnetic beads separate the hybrids from the supernatant. Non-specific sequences are washed away and the captured library is amplified, providing high read coverage around the targeted region. Strategies for targeted enrichment of total virome sequences have been deployed, resulting in promising capture platforms such as “VirCapSeq-VERT”, which is capable of detecting all vertebrate viruses with a detection limit comparable to that of real-time PCR [15]. Importantly, the detection of known pathogens can be accompanied by genomic reconstruction and the characterization of novel pathogens that present up to 40% genetic variability compared to the strains used as templates for the baits’ design [15] due to the partial alignment of the molecular baits to the target genomes (Figure 1). Moreover, target enrichment by hybridization enables the sequencing of both DNA and RNA viruses at the same time—after cDNA synthesis—by depleting the unwanted background genomic material. This approach is superior to standard poly-A selection, ribo-depletion and DNase treatment in terms of target selection specificity (Supplementary Table S1). Despite the great potential of these technologies, the extremely expensive equipment and reagents, the extended TAT and the need of dedicated laboratory infrastructures, still limit their real-time use in patient care, especially for PoC applications.

In 2014, Oxford Nanopore Technologies (ONT) announced a new, long-read, third-generation sequencing platform based on nanopore sequencing, the MinION. This USB-interfaced lighter-sized sequencer has been commercially available since 2015 and is nowadays able to produce more than 10 gigabases of data from a single flow cell, which comprises an array of 512 nanopores. The improved accuracy and the incomparable portability make MinION unique for PoC applications; for example, it has been successfully employed in the portable surveillance of the Ebola [16] and Zika [17] viruses. MinION is a very flexible device, but a major drawback remains the limited throughput of a single flow cell. As a result, it has low sensitivity when used for the detection of pathogens in the presence of a host’s genomic DNA. The platform has now reached a state of maturity to be used in the development of PoC diagnostic applications, at least as a proof-of-concept. By developing the first dedicated method for the targeted enrichment of MinION libraries, we have shown that this strategy can effectively enrich specific targets and their flanking genomic sequences [18,19]. Additionally, given the very special characteristic of MinION to produce extremely long reads, the mining of long and partially unknown genomic fragments from a complex nucleic acid mixture, is very important for the characterization of emerging pathogens with an unknown genomic sequence, for which standard PCR amplification assays cannot be designed. The presence of background genomic material can affect the sensitivity of metagenomic diagnostics in portable devices, such as MinION, due to their limited throughput. This is crucial, especially in the case of viruses, where the viral genome usually corresponds to less than 1% of the total genomic material present in the sample.

In this study, we examine the potential impact of targeted sequencing by hybridization on the unbiased detection and full genome reconstruction of both DNA and RNA viruses, in a single library preparation. We quantitatively evaluate the efficiency of the method in depleting various background genomic materials. Most importantly, we further explore the usage of the method in the characterisation of the, until recently, unknown virus SARS-CoV-2, which, intentionally, was not included in the baits’ design. This proof-of-concept approach highlights the potential usage of the method in the de novo identification of emerging human viruses in the future, using updated baits’ panels.

## 2. Materials and Methods

### 2.1. Sample Preparation

Mixed viral NGS libraries were derived from DNA or RNA isolated from eight clinical samples (nasopharyngeal swabs, urine, tissue, bronchoalveolar lavage and blood) already diagnosed positive for the presence of five RNA viruses, namely severe acute respiratory syndrome coronavirus 2 (SARS-CoV-2), coronavirus OC43 (HCoV-OC43), influenza A virus H3N2 (infA(H3N2)), respiratory syncytial virus (RSV) and human coxsackievirus A16 (Cox A16) and three DNA viruses, namely, cytomegalovirus (CMV), parvovirus B19 (B19V) and human adenovirus C (HAdV-C). Care was taken so that the RNA and DNA samples included in this study were anonymized prior to analysis in line with the EU General Data Protection Regulation (GDPR) mandates. Extraction of the viral genomic DNA and RNA was performed with the automated platform NucliSens-EasyMag (BioMérieux, Marcy l’Etoile, France) according to the manufacturer’s instructions. Quantification of the total RNA and DNA was performed on the Qubit fluorometer (ThermoFisher Scientific, Waltham, MA, USA), initially calibrated with the Qubit RNA HS Assay (declared assay range between 25–500 ng/mL; sample starting concentration between 250 pg/µL–100 ng/µL) and the Qubit dsDNA HS Assay (declared assay range between 1–500 ng/mL; sample starting concentration between 10 pg/µL–100 ng/µL). Equimolar concentrations of DNA/RNA viral extracts were mixed to be used as a template for the generation of targeted and untargeted NGS libraries.

The RNA pool was first subjected to reverse transcription and second strand synthesis using the Maxima H Minus Double-Stranded cDNA Synthesis Kit (ThermoFisher Scientific, Waltham, MA, USA). The DNA/RNA viral pool was then divided into four aliquots that were processed in parallel; two aliquots—one for the targeted and one for the untargeted library—were processed with the KAPA HyperPlus Library Preparation Kit (Roche Diagnostics, Basel, Switzerland), while the two others—one for the targeted and one for the untargeted library—were processed with the ONT SQK-LSK109 “Genomic DNA by Ligation” protocol (Oxford Nanopore Technologies, Oxford, UK) for sequencing on the MinION. Following the HyperCap Target Enrichment Kit (Roche Diagnostics, Basel, Switzerland) instructions, the VirCapSeq-VERT hybridization probes (kindly provided by Roche Diagnostics (Hellas)) were used for targeted virome enrichment, with ~two million probes covering the genomes of members of all viral taxa known (up to 2015) to infect vertebrates, including humans [15]. With reference to the MinION targeted library, the library preparation followed the target enrichment procedure due to the susceptibility of the protein-containing MinION adapters to temperature [18]. 

Performance of the virome target enrichment was verified by specific real-time PCRs for 3 viruses (Cox A16, HAdV-C, RSV), before and after the hybridization process. Similarly, depletion of the host genome was verified via real-time PCR against the ribonuclease P (RNase P) gene (data not shown). The normalised read depth was also estimated for each reference genome (Table 1). Both targeted and untargeted libraries were sequenced using the NextSeq500 platform (Illumina, San Diego, CA, USA) and the MinION sequencer (Oxford Nanopore Technologies, Oxford, UK).

### 2.2. Bioinformatics

Raw data were aligned on the complete reference sequences of the following viruses: SARS-CoV-2 (NC_045512.2), HCoV-OC43 (NC_006213.1), infA(H3N2) (the eight segments of the reference genome (NC_007366.1—NC_007373.1)), RSV (NC_001803.1), Cox A16 (U05876.1), CMV (NC_006273), B19V (NC_000883.2) and HAdV-C (NC_001405.1) using Bowtie2 [20] after quality check with FastQC (www.bioinformatics.bbsrc.ac.uk/projects/fastqc (accessed on 1 January 2021)). Bowtie2 alignment was also performed against the Homo sapiens (human) genome assembly GRCh37 (hg19) (RefSeq assembly accession: GCF_000001405.13), the human mitochondrial DNA (mtDNA) (NC_012920.1) and ribosomal DNA (rDNA) (U13369.1), and a dedicated full length 16S rRNA gene database downloaded from Greengenes [21]. All alignments were visualized with the Integrated Genomics Viewer (IGV) [22]. In order to simulate the reconstruction of the SARS-CoV-2 genome, de novo assembly was performed, using SPAdes assembler [23] for the Illumina target enriched dataset and Smartdenovo [24] for the MinION target-enriched dataset. Prior to the assembly, the raw data were aligned against the human genome reference GRCh37 in order to filter out the mapped reads. The remaining—unmapped—reads were assembled and the resulting contigs were aligned against the NCBI RefSeq viral database using BLAST [25]. Basic computations and visualizations were implemented in R programming language R version 3.6.2, using in-house scripts.

## 3. Results

### 3.1. Efficiency of Total Virome Target Enrichment

The objective of this study was to evaluate targeted total virome sequencing in both large-scale and portable platforms and to assess the efficacy of the method in detecting and characterizing unknown, emerging viruses such as SARS-CoV-2.

**Figure 2:**
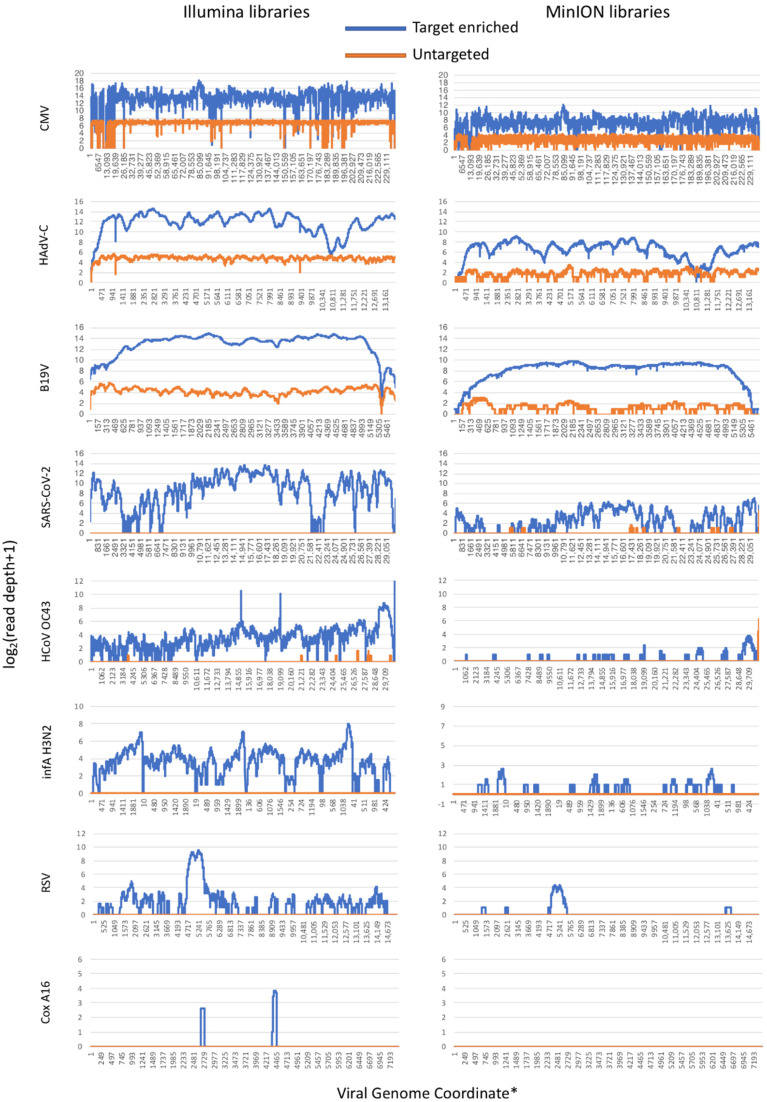
Read depth across viral genomes for the Illumina (**left**) and the MinION (**right**) experiments. Target-enriched libraries are in blue, while untargeted libraries are in orange. * Coordinates for the 8 genomic segments of infA(H3N2) virus are concatenated.

The untargeted Illumina sequencing run resulted in 32,235,720 single end reads, the majority of which (93.45%) mapped to the human genome, while only 0.94% in total were mapped to the viral genomes. The virome-targeted Illumina run resulted in 63,664,520 reads, with more than 83.03% on-target reads mapped to all viruses and only ~10% off-target reads, reflecting a substantial enrichment compared to the host background (Human gDNA, Human mtDNA, Human rRNA/DNA) and the bacterial DNA (16S gene). The efficiency of the enrichment was lower in the case of CoxsA16, as only 18 reads mapped to the reference sequence after the enrichment. It should be noted though, that Cox A16, along with infA(H3N2), RSV and SARS-CoV-2 remained undetected in the untargeted library. Most importantly, 797,191 reads were mapped on the SARS-CoV-2 reference genome in the targeted library, although no specific baits for this genome were included in the panel. The targeted library was enriched in both DNA and RNA viral genomes in contrast to the untargeted dataset, which contained reads only for the three DNA viruses, with the minor exception of nine reads mapping to the HCoV-OC43 genome. The genome coverage was substantially increased to more than 90% in all enriched viruses, with the exception of RSV (63.62%) and the unsuccessfully enriched Cox A16. (Table 1, Figure 2). The enrichment resulted in a more successful depletion of the human gDNA and the mtDNA (10-fold decrease), rather than human rRNA/DNA and the bacterial genomic material (Supplementary Figure S1).

For the MinION untargeted sequencing, the dataset contained 1,866,680 reads, while the targeted sequencing resulted in 1,129,865 reads. Only 0.6% of the untargeted reads mapped to the viral genomes, while the majority of them (70.86%) mapped to the human genome. On the contrary, the targeted library resulted in 35.41% reads mapping to the viral genomes, out of which 32.48% were on CMV and only 14.07% on the human genome. A substantial fold increase in the reads (%) mapping to SARS-CoV-2 (~4×), CMV (~60×), HAdV-C (~100×) and B19V (~300×) was observed. Similarly, genome coverage was found to be increased to more than 90% in the case of B19V (from 63.44% to 98.86%) and in HAdV-C (from 69.58% to 97.48%). SARS-CoV-2 genome coverage was also increased (from 4.18% to 74.46%), but near full genome reconstruction was not possible from this mapping alignment. The genome coverage was lower wherever the enrichment was moderate such as in the case of infA(H3N2) (from 0.00 to 25.72%), HCoV-OC43 (from 0.14% to 20.09%) and RSV (from 0.00 to 9.23%), allowing the detection of the viruses but not the complete reconstruction of their genomes. Cox A16 remained undetected in both the untargeted and targeted libraries. The reads (%) mapping to the human genome and the mtDNA were decreased five-fold. Only a moderate two-fold decrease in the human rRNA/DNA reads was reported, while the enrichment did not successfully deplete the bacterial DNA (Table 1, Supplementary Figure S1).

### 3.2. De Novo Assembly—Reconstruction of SARS-CoV-2

**Figure 3:**
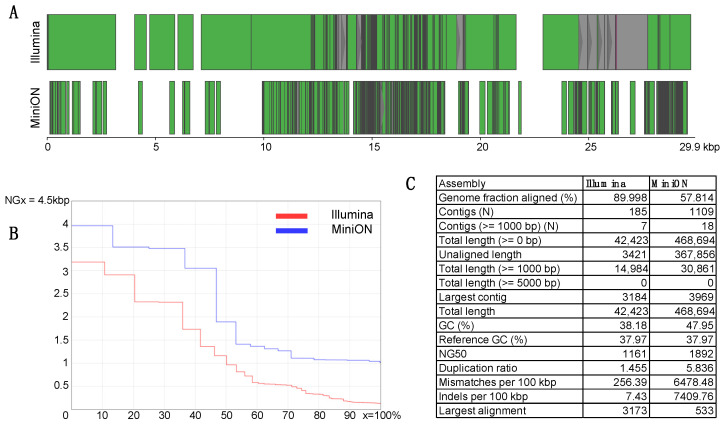
Comparison of SARS-CoV-2 de novo assemblies from Illumina and MinION target enriched libraries. (**A**) Alignment of Illumina (top) and MinION (bottom) contigs on the SARS-CoV-2 genomic map. Green contigs represent concordant alignments, while grey contigs represent discordant alignments. (**B**) Comparison of the NGx metric for Illumina (red) and MinION (blue) assemblies. (**C**) Comparative metrics of size and error profiles for both assemblies.

The efficient enrichment of SARS-CoV-2 sequences in both the Illumina- and the MinION-targeted libraries enabled the de novo reconstruction of contigs covering 89.99% and 57.18% of the viral genome, respectively (Figure 3). It should be mentioned though that the assessment of the assemblies revealed better metrics for MinION with regard to the sizes of the resulting contigs and the NG50 (1161 for Illumina vs. 1892 for MinION). Conversely, although the total length of the assembly was 10-fold higher for MinION, the substantially higher number of mismatches per 100 kbp (6478.48 vs. 256.39) and indels per 100 kbp (7409.76 vs. 7.43) reflect a profile with longer, yet erroneous contigs, which do not align with the reference sequence, resulting in lower overall coverage. The erroneous profile of the MinION de novo assembly is also reflected in the unaligned fraction, which exceeds 78.5% (368,756 bp out of 468,694 bp), in contrast to only 8% of unaligned assembly in the case of the Illumina dataset. 

## 4. Discussion

The detection and characterization of viruses in clinical samples can be challenging, mainly because of the low viral titer and the complex combination of a viral and a robust host genomic background. Even though immense advances in NGS technologies have led to the development of various experimental protocols, the time and cost required to sequence large, complex libraries in high read depth has not yet been overcome [26,27]. Hence, a committed effort has been devoted in order to develop target-enrichment methods to capture selected genomic regions of interest.

Efficacy of the sequence capture is expressed by the extent to which the target regions are enriched, calculated as the proportion of total reads derived from the region of interest divided by the reads derived from the untargeted regions [28]. Applications of targeted enhancement can lead to a dramatic increase in sensitivity for virus detection, exceeding methods such as amplicon sequencing [29]. The approach of target enrichment offers a solution more feasible than typical PCR and its variations, as it ensures that non-specific amplification due to interaction between multiple primer pairs will not take place, while it overcomes issues, such as the upper limit to the amplicon’s length generated and the normalization of the products’ concentration [30]. Targeted enrichment has been shown to increase the sensitivity of detection and to enhance the full genome sequencing of the dengue 1–4, chikungunya and Zika viruses in clinical samples with low viral loads [14] For example, in SARS-CoV-2 positive samples with low viral copy number, the positivity of the infection is confirmed by the amplification of orf1ab and 11 genes, which are related to virulence and the analysis of the sequencing output regarding the read number, the depth and sequencing coverage and the identity [31]. 

Target-enrichment methods are under continuous development, in order to achieve higher sensitivity with reference to the detection of mutations and the characterization of structural variability, in terms of insertions and deletions [26]. The elimination of genomic—background—DNA, allows for a higher-depth NGS analysis of the remaining targeted sequences, thus enabling the detection of single nucleotide polymorphisms (SNPs) and copy number variants (CNVs) in clinical samples, as well as the retrieval of pathogen nucleic acids and the reconstruction of known and unknown genomes derived from infectious agents found in low abundance within the host [26,32]. This was confirmed in our study, as the sequencing output of the untargeted libraries revealed the presence of less than 1% of total viral reads in the datasets, while, specifically, respiratory RNA viruses such as infA(H3N2), RSV and Cox A16 remained undetected. Conversely, when targeted virome enrichment, based on the “VirCapSeq-VERT” strategy for large-scale and portable platforms, was applied prior to the sequencing analysis, the percentage of the total reads reported as viral in targeted libraries was 0.004% and 0.0028% for infA(H3N2) in the Illumina and the MinION runs, respectively. The percentage of total reads mapped on the CMV genome was 0.94% and 0.5% in the Illumina and the MinION sequencing runs of the untargeted libraries, while it increased to 76.48% and 32.48%, respectively, after the target enrichment with VirCapSeq-VERT, the platform which is known to result in a 100- to 1000-fold increase in viral genomes from blood [15]. Importantly, both the Illumina- and MinION-enriched libraries presented a higher capacity in mining viral sequences originating from RNA viruses. According to our results, following an enrichment- or amplification-based protocol prior to high throughput sequencing, would enhance the detection of RNA viruses since they remained, in most cases, undetected in the untargeted libraries [33,34].

In our study, we utilized the VirCapSeq-VERT strategy for the detection of the novel virus SARS-CoV-2, although the corresponding reference genome was not included in the initial design of the baits’ panel. The baits hybridize to short conserved sequences, presenting genomic variation up to 40% [15]. The successful detection and reconstruction of SARS-CoV-2 reported in this study (96.12% coverage of the reference genome for the Illumina mapping alignment), is a proof-of-concept, indicating that partial alignment of the molecular baits to the viral genomes can enable the detection and typing of unknown, emerging human viruses. As highlighted by the recent pandemic, false negative diagnostic results (e.g., during PCR screening) may have grave consequences and it becomes more important in cases where the sensitivity of the diagnostic assays can be compromised by mutations accumulated in regions that correspond to PCR assay targets (primers and probes annealing regions) [35]. Thus, technical advancements improving detection sensitivity via unbiased methods based on NGS are urgently needed. Especially in the context of emerging viruses, where the reference genome may not be available, enrichment allowing the de novo reconstruction of the genome is very useful. In this study, we deployed a simulating analytical pipeline to de novo reconstruct the SARS-CoV-2 genome from both Illumina and MinION total virome-enriched datasets. Our results also highlight the importance of the NGS platform accuracy with regard to the completeness of the assembly. Although the MinION-generated reads successfully covered the majority of the genome in the mapping alignment (using the reference as a guide), the de novo assembled contigs, based on the same reads, could only cover ~58% of the genome. These contigs, however, were substantially larger compared to those generated from the Illumina reads. Taken together, these results support the conclusion that MinION datasets can be very useful in the reconstruction of unknown or highly repetitive viral genomes since they largely contribute in their phasing, especially in combination with other NGS platforms generating shorter, yet more accurate reads [36,37]. 

Emerging and re-emerging zoonotic infectious diseases pose a great threat to public health, presenting a great increase in incidence, varying by geographic region and population [38] with the ease of their transmission being facilitated by intercontinental travel [39], while their epidemiology is markedly affected by the complex host–pathogen interaction [38]. In a globalised world, trade across international borders facilitates the spread of pathogens, and therefore specific counter measures are needed to rapidly control these spreads [11]. Consequently, there is great importance in the investigation of the precision, robustness and applicability of diagnostic platforms such as the portable sequencer MinION for PoC applications, allowing the effective and prompt containment of the spread, thereby limiting a subsequent global outbreak. However, many of the MinION library preparation steps, and especially the hybridization procedures deployed in our study, still need standard laboratory equipment, which restricts their applicability in the field. Further development of dedicated microfluidic devices that would integrate parts of the workflow would largely simplify these complex procedures. 

Our study highlights the importance and the usability of total virome target enrichment, using both large-scale and portable NGS platforms. In the context of a prospective study, extensive testing using undiagnosed clinical specimens and comparison with real-time PCR diagnostics will facilitate the standardization of this method towards the development of a diagnostic platform for the detection and identification of emerging viruses with increased accuracy.

## 5. Conclusions

Targeted virome sequencing enhances the simultaneous detection of multiple DNA and RNA viruses extracted directly from clinical samples. The depletion of the background genomic material varies depending on its source. The method can be potentially used for the identification of unknown, emerging human viruses, which may differ from the viral strains used for the design of the baits’ panel. 

## Figures and Tables

**Table 1:** Comparison of mapping statistics for untargeted and targeted libraries.

	Illumina				MinION(ONT)			
	Untargeted/Targeted				Untargeted/Targeted			
Total Reads(N)	32,235,720/63,664,520				1,866,680/1,129,865			
	Mapped Reads (N)	Mapped Reads (%)	Genome Coverage (%)	Read Depth (X, Mean)	Mapped Reads, N	Mapped Reads (%)	Genome Coverage (%)	Read Depth (X, Mean)
**Viral Genome**								
CMV	295,623/48,693,256	0.9171/76.4841	96.71/97.38	17,873.30/20,176.00	9522/367,035	0.5101/32.4849	95.58/96.81	8.09/310.59
HAdV-C	8549/2,438,953	0.0265/3.8309	88.46/92.83	23.42/6617.77	348/22,324	0.0186/1.9758	69.58/97.48	2.51/154.86
B19V	1129/726,199	0.0035/1.1405	99.76/99.98	19.88/12,782.60	38/7620	0.0020/0.6744	63.44/98.86	1.84/433.58
SARS-CoV-2	0/797,191	0.0000/1.2522	0.00/96.12	0.00/1800.46	1285/2974	0.0688/0.2632	4.18/74.46	1.53/14.39
HCoV-OC43	9/141,470	0.0041/0.2222	2.44/96.07	0.03/152.40	76/102	0.0041/0.0090	0.14/20.09	0.08/0.54
infA-H3N2	0/2733	0.0000/0.0043	0.00/93.87	0.00/19.40	0/32	0.0000/0.0028	0.00/25.72	0.00/0.45
RSV	0/4119	0.0000/0.0065	0.00/63.62	0.00/24.90	0/49	0.0000/0.0043	0.00/9.23	0.00/0.61
Cox A16	0/18	0.0000/0.0000	0.00/2.64	0.00/0.21	0/0	0.0000/0.0000	0.00/0.00	0.00/0.00
**Background**								
Human Genome (hg19)	30,125,987/6,016,007	93.4553/9.4495			1,322,818/158,985	70.8647/14.0712		
Human mtDNA	62,049/12,196	0.1925/0.0192			3461/371	0.1854/0.0328		
Human rRNA/DNA	228,575/501,020	0.7091/0.7870			44,473/13,166	2.3825/1.1653		
16S DNA	11,444/53,525	0.0355/0.0841			1456/1172	0.0780/0.1037		

## Data Availability

Not applicable.

## Supplementary Materials

The following supporting information can be downloaded at: https://www.mdpi.com/article/10.3390/v14061272/s1, Supplementary Table S1: Comparison of virome enrichment strategies. Supplementary Figure S1: Relative fold of enrichment for viral sequences compared to background genomic material.
