# Supplementary material for: Targeted Virome Sequencing Enhances Unbiased Detection and Genome Assembly of Known and Emerging Viruses—The Example of SARS-CoV-2
Source: Viruses. 2022 Jun 11;14(6):1272. doi: 10.3390/v14061272 (PMC9227943; doi:10.3390/v14061272)
Supplement: Supplementary file 1 [file viruses-14-01272-s001.zip › viruses-1761915-supplementary Table S1.pdf]

Supplementary Table S1. Comparison of virome enrichment strategies.

	Total Virome Enrichment Method [1]			
	poly-A selection	ribo-depletion	DNAse treatment	Target Enrichment
Viral Target				
viral genome - DNA	-	+	-	+
viral genome - RNA -strand	-	+	+	+
viral genome - RNA +stand	+	+	+	+
viral transcripts -polyA	+	+	+	+
viral transcripts -non polyA	-	+	+	+
Background NAs [2]				
Human gDNA	-	+	-	-
Human rRNA	-	-	+	-
Human mRNA	+	+	+	-
bacterial gDNA	-	+	-	-
bacterial rRNA	-	+	+	-
bacterial mRNA	-	+	+	-

[1] Plus (+)indicates the substantial presence of the genomic material in the final NGS library.
[2] Major sources of background genomic material in a clinical specimen. Other sources like mitochondrial transcripts, miRNAs, long-non-coding RNAs, other microorganisms and parasites etc. are omitted.
