# Supplementary figures and images for: Targeted Virome Sequencing Enhances Unbiased Detection and Genome Assembly of Known and Emerging Viruses—The Example of SARS-CoV-2
Source: Viruses. 2022 Jun 11;14(6):1272. doi: 10.3390/v14061272 (PMC9227943; doi:10.3390/v14061272)

## Illumina

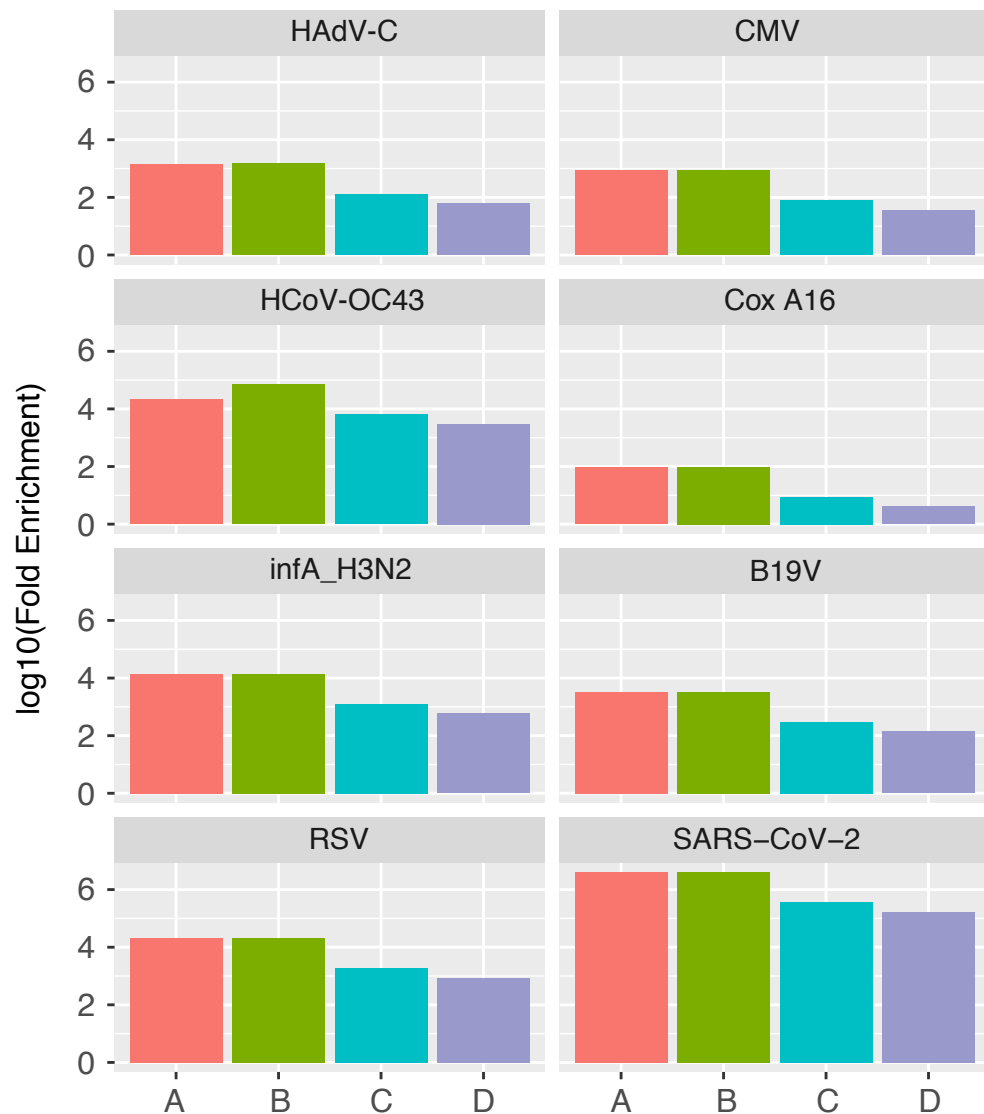

## MinION

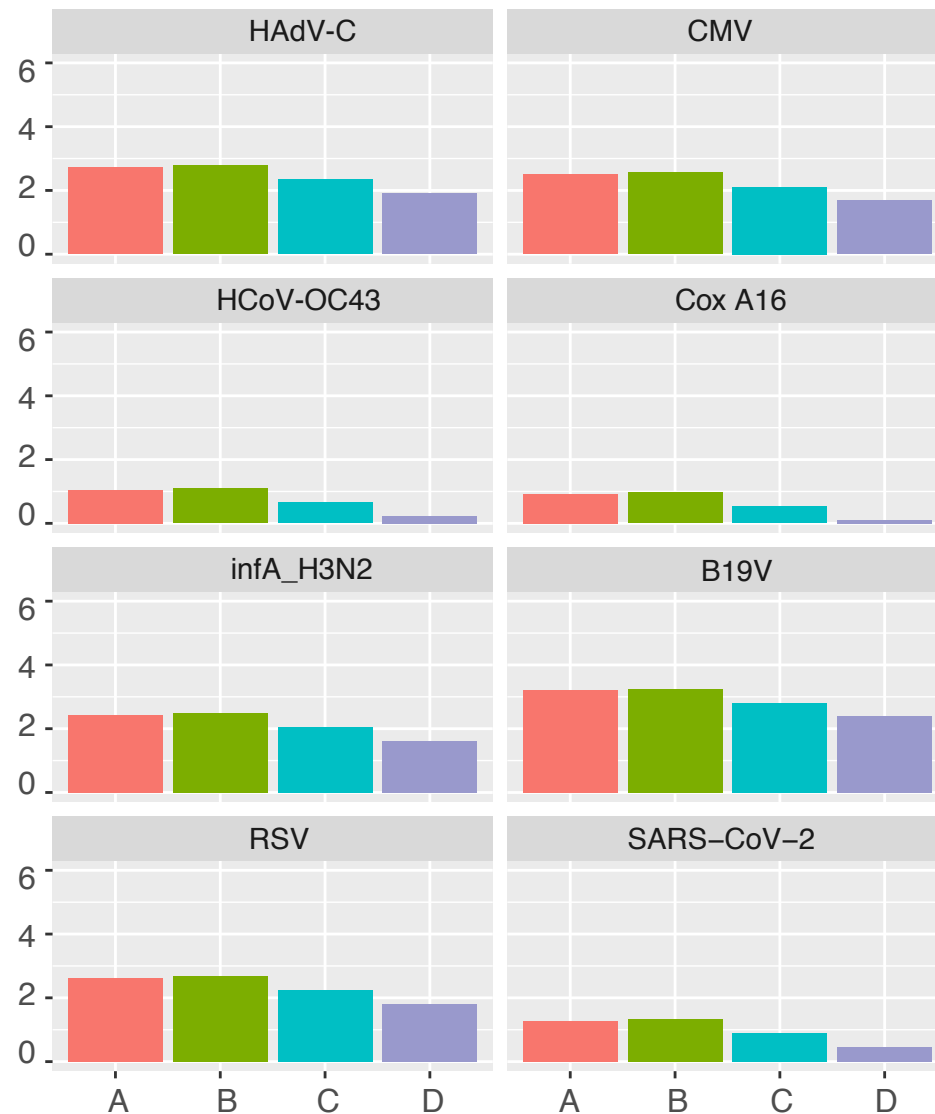

key

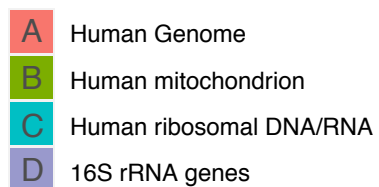

Supplement: Supplementary file 1 [file viruses-14-01272-s001.zip › viruses-1761915-supplementary Figure S1.pdf]
